# Differential Hyperphosphorylation of Tau-S199, -T231 and -S396 in Organotypic Brain Slices of Alzheimer Mice. A Model to Study Early Tau Hyperphosphorylation Using Okadaic Acid

**DOI:** 10.3389/fnagi.2018.00113

**Published:** 2018-04-19

**Authors:** Bettina M. Foidl, Christian Humpel

**Affiliations:** Laboratory of Psychiatry and Experimental Alzheimer’s Research, Medical University of Innsbruck, Innsbruck, Austria

**Keywords:** okadaic acid, Alzheimer’s disease, tau phosphorylation, wortmannin, organotypic brain slices, transgenic mice

## Abstract

Alzheimer’s disease (AD) is a progressive neurodegenerative disorder of the brain, characterized by extracellular aggregation of beta-amyloid (Aβ) and hyperphosphorylation of tau causing intraneuronal neurofibrillary tangles (NFTs). There is urgent need to study the interactions between Aβ and tau, especially to solve the question of the pathological cascade. In the present study, we aim to develop a model of organotypic brain slices in which both plaque and tau pathology can be examined. Organotypic brain slices (150 μm thick, coronal, at the hippocampal level) from adult (9 month) wildtype (WT, C57BL/6N) and transgenic AD mice (TG, APP_SweDI) were cultured for 2 weeks. To induce tau hyperphosphorylation 100 nM okadaic acid (OA), 10 μM wortmannin (WM) or both were added to the slices. Hyperphosphorylation of tau was tested at tau-S199, tau-T231 and tau-S396 using Western blot. Our data show that in TG mice with plaques a 50 kDa fragment of tau-S396 was hyperphosphorylated and that OA induced hyperphosphorylation of tau-S199. In WT mice (without plaques) OA caused hyperphosphorylation of a 50 kDa and a 38 kDa tau-T231 form and a 25 kDa sdftau-S396 fragment. The N-methyl-D-aspartate (NMDA) antagonist MK801 (1 μM) did not block these effects. Immunohistochemistry showed diffuse increased tau-S396 and tau-T231-like immunoreactivities at the hippocampal level but no formation of NFTs. Confocal microscopy indicated, that pTau-T231 was preferentially located in cytoplasma surrounding nuclei whereas pTau-S396 was found mainly in nerve fibers and strongly associated with plaques. In conclusion we provide a novel *in vitro* model to study both plaque and tau hyperphosphorylation but not NFTs, which could be useful to study pathological processes in AD and to screen for drugs.

## Introduction

Alzheimer’s disease (AD) is the most common form of dementia worldwide but the underlying mechanisms leading to AD are still not fully understood. Two major hallmarks appear to be characteristic for AD: the accumulation of extracellular beta-amyloid (Aβ) plaques and the hyperphosphorylation of tau leading to the formation of intraneuronal neurofibrillary tangles (NFTs; Loeffler et al., 2015; Ahn et al., 2016). The most prominent hypothesis for AD is the Aβ cascade, where a successive cleavage of the amyloid precursor protein (APP) and an imbalance between the clearance and production of Aβ leads to an abnormal accumulation of Aβ in the brain. It is suggested that Aβ triggers a neurodegenerative cascade of inflammation and the formation of NFTs including neuronal death (Turner et al., 2003; Gandy, 2005; Barage and Sonawane, 2015; Herrup, 2015). However, the hypothesis also has limitations as the interplay between plaques, the hyperphosphorylation of tau with the formation of NFTs and the exact initiation of the cascade is still not fully understood (Haass and Mandelkow, 2010; Reitz, 2012). Though, some studies give rise to the direction that an accumulation of Aβ leads to the deposition of tau (Bateman et al., 2006; Selkoe and Hardy, 2016). Studies about the formation of mutated tau show in contrast that e.g., in frontotemporal dementia formation of tangles is existent without the deposition of Aβ (Jin et al., 2011; Selkoe and Hardy, 2016).

The central question about the role of tau in the Aβ cascade, especially whether if Aβ drives the tau pathology or vice versa or if both pathologies have synergistic effects has to be proven. It is well known that tau is a microtubule-associated protein (MAP) and physiologically stabilizes and regulates the microtubule structure by controlling axonal diameters and axonal transport (Hanger et al., 2009b; Ward et al., 2012; Luna-Munoz et al., 2013; Wang and Mandelkow, 2016). However, the physiological roles of tau seem to be more complex and by far not fully explored. Tint et al. (1998) showed that an acute inactivation of tau in cultured sympathetic neurons had no effect on the dynamics of microtubules. Similarly, Ikegami et al. (2000) argued that in tau-deficient mice axonal elongation of cultured neurons was not affected, but microtubule stability was decreased in some small caliber axons (Harada et al., 1994). Yuan et al. (2008, 2013) found that axonal transport rates are not affected by a deletion or overexpression of tau in mice, which means that tau is not necessarily essential for the axonal transport (Yuan et al., 2008). In addition there is also the assumption that tau plays a pivotal role in targeting dendrites via activation of the kinase Fyn (tau hypothesis). In this regard, it is possible that the toxicity of Aβ is linked to dendritic tau (Haass and Mandelkow, 2010; Ittner et al., 2010; Ittner and Götz, 2011). It is currently extensively discussed if tau becomes hyperphosphorylated under pathological conditions leading to the formation of intracellular filament-shaped aggregates in the neuronal cell body (Trojanowski et al., 2005; Li et al., 2006; Patterson et al., 2011; Ward et al., 2012).

There is urgent need to study the interplay between Aβ toxicity and tau. The interaction could be studied in triple transgenic (TG) AD mice, where plaques and tau pathology is present. However, such *in vivo* models have many limitations. First, very old animals (approximately 15–20 months) need to be analyzed, which is tricky and expensive. Second, such models only partly represent the human situation. And third, the cascade of events (first Aβ and then tau or vice versa) cannot be easily tested. Thus, potent *in vitro* models need to be developed. We recently developed a novel *in vitro* model of adult organotypic brain slices taken from 9-month-old AD mice (Humpel, 2015b). Using such an organotypic brain slice model of adult mice we demonstrated elimination of Aβ plaques using Aβ degrading enzymes (Humpel, 2015b). However, in this model only Aβ plaques are found and the tau pathology is missing. Thus, we are highly interested to develop a more complex *in vitro* model where plaques as well as tau pathology is seen.

In our present study we used organotypic brain slices of wildtype (WT) and transgenic (TG) AD mice and aimed to examine the effects of different treatments which may lead to an increased hyperphosphorylation of tau. We will use okadaic acid (OA) or wortmannin (WM) to induce hyperphosphorylation of tau at three tau phosphoepitopes (tau-S199, tau-T231 and tau-S396).

## Materials and Methods

### Animals

Nine-month-old WT (C57BL/6N) and TG APP_SweDI (SweDI; expressing APP harboring the Swedish K670N/M671L, Dutch E693Q, and Iowa D694N mutations; C57BL/6-Tg(Thy1-APPSwDutIowa)BWevn/Mmjax) mice were purchased from MMRRC (USA). These mice are fully characterized and develop plaques at the age of 5–6 months (Davis et al., 2004). Mice are housed at the Innsbruck Medical University animal facility providing open access to food and water under 12/12-h light-dark cycles. All experiments were approved by the Austrian Ministry of Science and Research and conformed to the Austrian guidelines on animal welfare and experimentation.

### Organotypic Brain Slices and Vibrosections

Adult mice were rapidly sacrificed and the head quickly transferred in 70% ethanol, the brains dissected and glued (Glue Loctite) onto the chuck of a water cooled vibratome (Leica VT1000A) and triggered close to a commercial shave racer. Under aseptic conditions, 150 μm thick vibrosections were cut and collected in sterile medium. The organotypic vibrosections were carefully placed onto a 0.4 μm membrane insert (Millipore PICM03050) within a 6-well plate. Vibrosections (2 per well) were cultured in 6-well plates (Greiner) at 37°C and 5% CO_2_ with 1 ml/well of the “Slice culture medium” (horse serum 10%, MEM-Hepes, NaHCO_3_, Glucose, Hank’s Solution, Antibiotikum, Glutamine) for 2 weeks. To induce hyperphosphorylation OA (100 nM; Santa Cruz, sc-3513) or WM (10 μM, Sigma Aldrich, w1628) or combinations were added to the medium. As these substances were dissolved in Dimethylsulfoxide (DMSO; Merck, 102952) control sections were incubated with respective DMSO equivalents. In selected experiments the N-methyl-D-aspartate (NMDA) antagonist MK801 (1 μM) was added to the slices with or without OA.

### Hyperphosphorylation of Recombinant Human and Mouse Tau

In order to perform positive controls for hyperphosphorylation of tau, 1 μg recombinant human tau (tau441, 2N4R, Covance PTN-5272) or mouse tau (residues Ala92-Val400; Cloud-Clone Corp, catnr. RPB983Mu01) was incubated with 2 μl glycogensynthase-kinase-3β (GSK-3β) stock (170–200 nmol min/mg, Sigma G4296) in 25 μl tau kinase buffer (40 mM HEPES, 5 mM EGTA, 3 mM MgCl2, pH 7.6) including 2 mM ATP overnight at 37°C. As a negative control either the enzyme or the protein was omitted. Ten μl of these reactions were tested by Western blot analysis.

### Confocal Microscopy

Confocal microscopy was performed using SP5 confocal microscope (3D images; Leica Microsystems, Wetzler, Germany) with an HCX PL APO ×63 and/or 1.3 NA glycerol objective. Images were acquired using the LAS AF acquisition software, version 2.1, and further processed with Huygens Deconvolution and Imaris 8.1 Image management software. Confocal imaging was performed with an argon laser line (set power to 30%) for AlexaFluor 488, a DPSS561 nm laser for AlexaFluor 546 or Thiazine Red and a 405 diode laser for DAPI. Emission of each fluorophore was detected from 493 nm to 556 nm (AlexaFluor 488, eGFP), 566–628 nm (AlexaFluor 546, Thiazine Red) and 418–483 nm (DAPI). For the control panel the smart gain was set to 250 Volt (V) per turn, smart offset to 0.1 or 1%, zoom to medium, X position to fine, Y position to fine and the resolution was set to 8-bit, pixels size between 40 and 60 pixels, speed to approximately 1000 Hertz (Hz), frame resolution to 1024 × 1024 and the line average between 1–3. General parameters for the sampling intervals was set to X (nm) 60.125, Y (nm) 60.125, Z (nm) 125.885. For the objective correction the Photomultiplier (PMT) was activated and set to a gain of 500–600 V and the Scan Mode from XYZ to XZY. Afterwards AOBS was clicked and the setting changed to Reflection. The PMT detector range was set to min 487 nm and max 556 nm and “between lines” in the scanning mode. For the Deconvolution with the Huygens software the following parameters were set: numerical aperture (1.3), objective quality (good), coverslip position (μm), imaging directions (upward), lens immersion (glycerine, 1.474), embedding (Glyc. 90%, Mowiol, 1.458), backprojecting pinhole (307.09 nm), excitation fill factor (2.00), signal/noise per channel (15,15,15), max iterations (100), the search for background (auto), the background per channel (0.0, 0.0., 0.0), bleaching correction (if possible), brick mode (auto), quality change threshold (0.1%), iteration mode (optimized) and padding mode (automatic). After the Deconvolution the images were processed with the Imaris 8.1 software for 3D imaging.

### Viability Assay

Propidiumiodide (PI) staining was used to test the viability of slices. Two weeks cultured brain slices were freshly incubated with 2 μg/ml PI, then washed in phosphate buffered saline (PBS), and immediately visualized under the fluorescence microscope. Lactate dehydrogenase (LDH) release was performed as described by the manufacturer (Roche). As a control slices were incubated overnight with 2% Triton or for 3 days (2×/day) with 10 mM H_2_O_2_.

### Western Blot Analysis

Western blot analysis was performed as previously described by us (Hohsfield et al., 2014). Slices were scraped from the wells and collected into Eppendorf tubes. Subsequently, the samples were dissolved in 100 μl ice-cold PBS containing a protease inhibitor cocktail (Sigma Aldrich, P-8340) and a phosphatase inhibitor cocktail 1 (Sigma Aldrich, P0044-1 ML) plus phosphatase inhibitor cocktail 2 (Sigma Aldrich, P5726-1 ML). Slices were then sonicated using an ultrasonic device (Hielscher Ultrasonic Processor, Germany), centrifuged at 14,000× *g* for 10 min at 4°C. Afterwards the supernatant was used for analysis and 20 μl of the extracts were denatured (10 min, 70°C) and were loaded onto 10% Bis-Tris SDS-polyacrylamide gels (Thermo Fisher Scientific), separated for 35 min at 200 Volt (V) and finally electro-transferred to nylon-PVDF Immobilon-PSQ membranes for 20 min at 30 V in 20% methanol blotting buffer. Briefly, blots were blocked for 30 min in blocking buffer, incubated with primary antibodies against total tau (Tau-5; 1:1000, Thermo Fisher, AHB0042), phospho-tau-S199 (1:1000, ThermoScientific, 701054), phospho-tau-T231 (1:1000, BioLegend, 807201), phospho-tau-S396 (1:10,000, BioLegend, 807401), Neurofilament-200 kDa (1:10,000, Novus NB300–135) or actin (1:1000, Sigma Aldrich A2066) at 4°C overnight, washed, and then incubated in alkaline phosphatase conjugated anti-rabbit IgG for 30 min. After washing, bound antibodies were detected using an enhanced chemiluminescence (ECL) system and visualized by using a cooled CCD camera (SearchLight; Thermo Fisher Scientific). As a control a Western Blot (antibody Aβ 1–16 (6E10), Covance) was performed for aggregated Aβ from TG mice as reported earlier by us (Marksteiner and Humpel, 2008).

### Immunohistochemistry

Slices were postfixed and immunohistochemistry was performed as described by us (Kniewallner et al., 2016). Briefly, brain slices were washed with PBS and incubated in PBS/0.1% Triton (T-PBS) for 30 min at room temperature (RT) while shaking. After incubation, the sections were blocked in T-PBS, 20% horse serum (Gibco Invitrogen) for 30 min at 20°C while shaking. Following blocking, slices were incubated with 5 μl/well sample reducing agent (Invitrogen-Life tech, Vienna, Austria) for 10 min at 70°C cooled on ice and then incubated with the primary antibody tau-5 (1:250, Thermo Fisher, AHB0042), neurofilament-200 kDa (1:1000, Novus) and PHF Tau (p-Tau 202) clone AT8 antibody (1:500, Thermo MN1020) as well as phospho-tau-T231 (1:250, BioLegend, 807201; phospho-tau-S396, 1:250, BioLegend, 807401), in T-PBS and 0.2% bovine serum albumin (BSA) for 2–3 days at 4°C. The sections where then washed and incubated with anti-rabbit fluorescent Alexa 488 antibody (1:400, Invitrogen-Life Tech, Vienna, Austria) in T-PBS and 0.2% BSA for 1 h at 20°C while shaking. Finally the sections were washed and then mounted onto glass slides and coverslipped with Mowiol^®^ 4-88 (Roth, Austria). Alternatively, sections were stained with Thiazine Red (1.6 μg/ml, Sigma, overnight) to label plaques and counterstained with DAPI (1:10,000, 1 h) to visualize nuclei.

### Counting of Phospho-Tau Like-Immunoreactivity

Brain slices of TG mice treated with OA for 2 weeks were stained for p-tau T231, p-tau S396 (both Alexa-488), Thiazine Red and DAPI. The immunoreactivity for p-tau T231 and p-tau S396 was evaluated with the SP5 confocal microscope. Five fields (142 mm^2^) per slice were counted at a 10× magnification in a blinded way for each p-tau T231 and p-tau S396 and co-localized to Thiazin red and DAPI. The staining was counted in: (i) nerve fibers; (ii) cytoplasma surrounding DAPI+ nuclei; and (iii) their association with Thiazine red+ plaques.

### Data Analysis and Statistics

All data are reported as mean ± SEM optical density (OD). Western blots were normalized to actin expression and background was subtracted. Statistical analysis was performed with Student’s *T* Test and differences between mean values were determined using one-way ANOVA followed by a Fisher least significant difference *post hoc* test. Statistical results were considered significant at *p* < 0.05.

## Results

### Viability of Adult Brain Slices

**Figure 1:**
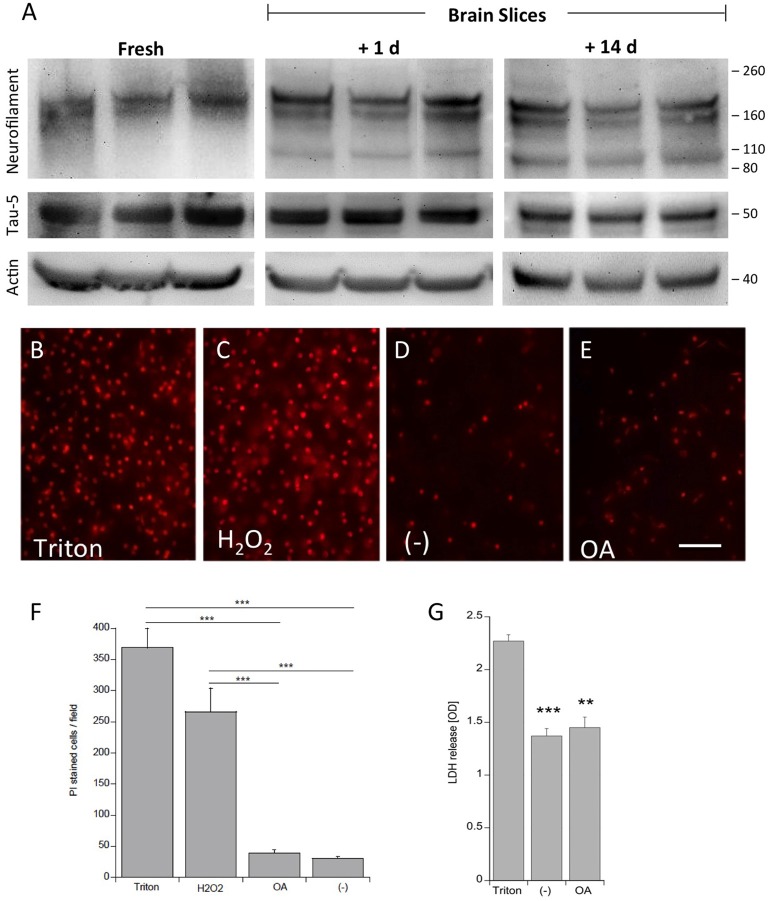
Proof of viability of organotypic adult brain slices. Brain slices of adult 9 month old wildtype (WT) mice were dissected fresh or incubated for either 1 day or for 14 days to examine vitality of slices **(A)**. Brain slices were subsequently analyzed by Western blot using antibodies against Neurofilament-200 kDa, total tau (Tau-5) and actin served as a control. Size markers are given as kDa on the right side. Note some degradation of neurofilament but stability of Tau-5 **(A)**. The viability of the slices was tested using propidiumiodide (PI) nuclear staining. Slices incubated with 1% Triton overnight **(B)** or 10 mM H_2_O_2_ for 3 days **(C)** gave very high positive staining, while slices incubated with **(E)** or without **(D)** okadaic acid (OA) only showed a few PI+ nuclei. The quantitative analysis shows the number of PI+ nuclei per field (697 mm^2^) **(F)**. Lactate dehydrogenase (LDH) release was measured in medium, showing high release in Triton-incubated slices and significantly reduced release in slices incubated with or without OA **(G)** Values are given as mean ± SEM (*n* = 3). Statistical analysis was performed by one-way ANOVA with a Fisher LSD *post hoc* test (***p < 0.001; **p < 0.01). Scale bar in **(E)** = 157 μm.

Viability of the slices was tested using neurofilament-200 kDa and tau-5 total staining in Western blot analysis (Figure 1). Fresh frozen slices showed a strong band for neurofilament-200 kDa, but a smaller neurofilament 110 kDa band became visible in slices incubated for 1 day and more pronounced for 14 days. Total tau was clearly visible at a 50 kDa size, however, was stable for 14 days in culture compared to fresh slices (Figure 1). Actin served as a control and was not affected (Figure 1). In addition, our data show that at the cellular level the brain structures were well preserved using DAPI nuclear staining (Figures 6A,B). Further, there was also a clear immunostaining for neurofilament and tau 5 in the dentate gyrus (DG; Figures 6G–J) and in the cortex (not shown), while omission of the primary antibody was negative (Figures 6E,F). PI was used to test the viability: while treatment of slices with 2% Triton or 10 mM H_2_O_2_ gave very high nuclear PI staining, the slices incubated with or without OA for 2 weeks displayed only few PI+ nuclei (Figures 1B–F). Release of LDH was markedly increased in Triton-incubated slices, and significantly reduced in slices incubated with or without OA (Figure 1G).

**Figure 2:**
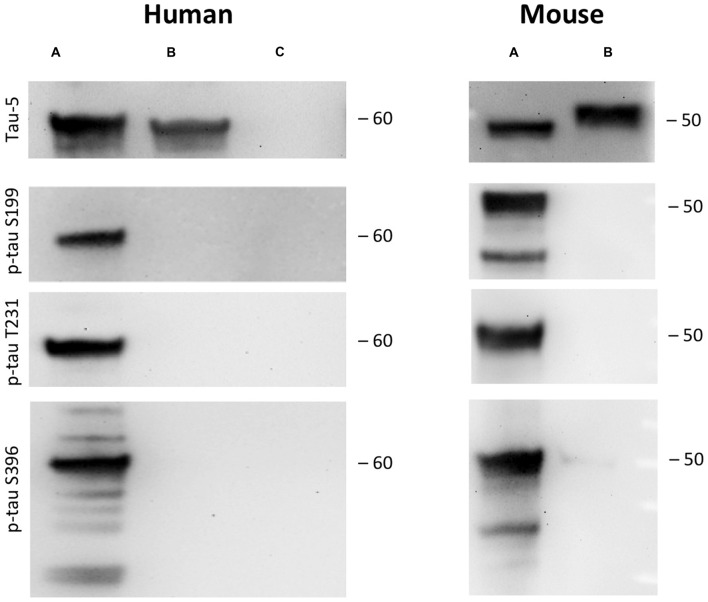
Tau phosphorylation *in vitro*. One microgram recombinant human tau (left, A) or mouse tau (right, A) was incubated with glycogensynthase-kinase-3β (GSK-3ß) and ATP in kinase buffer and analyzed by Western blot using antibodies against total tau (tau-5) phospho-tau-S199, phospho-tau-T231 and phospho-tau-S396. Lanes B and C served as a control omitting either the enzyme GSK-3β (Lane B) or tau protein (Lane C). Size markers are given as kDa.

**Figure 3:**
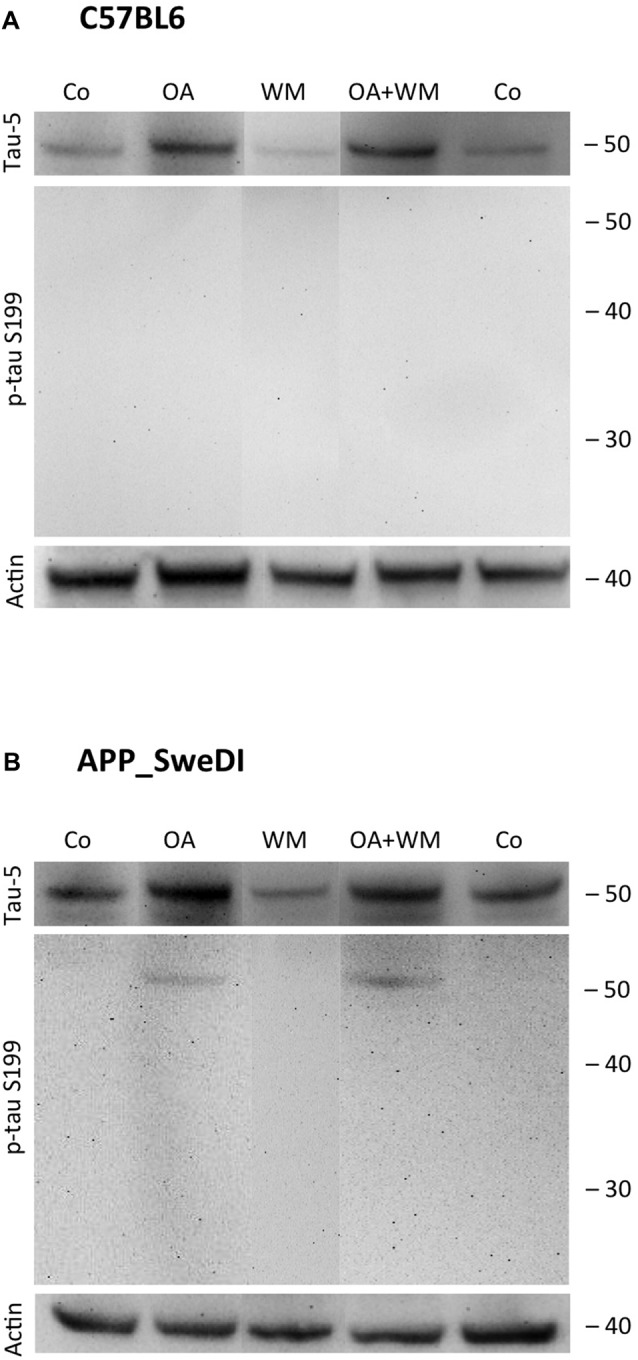
Western blot for p-tau S199. Brain slices of adult WT mice **(A)** or transgenic (TG) mice **(B)** were incubated for 14 days at 37°C without (Co) or with Okadaic acid (OA), wortmannin (WM), or combination of OA+WM. Extracted brain slices were subsequently analyzed by Western blot using antibodies against total tau (tau-5) and phospho-tau-S199. Size markers are given as kDa on the right side. Actin served as a control.

**Figure 4:**
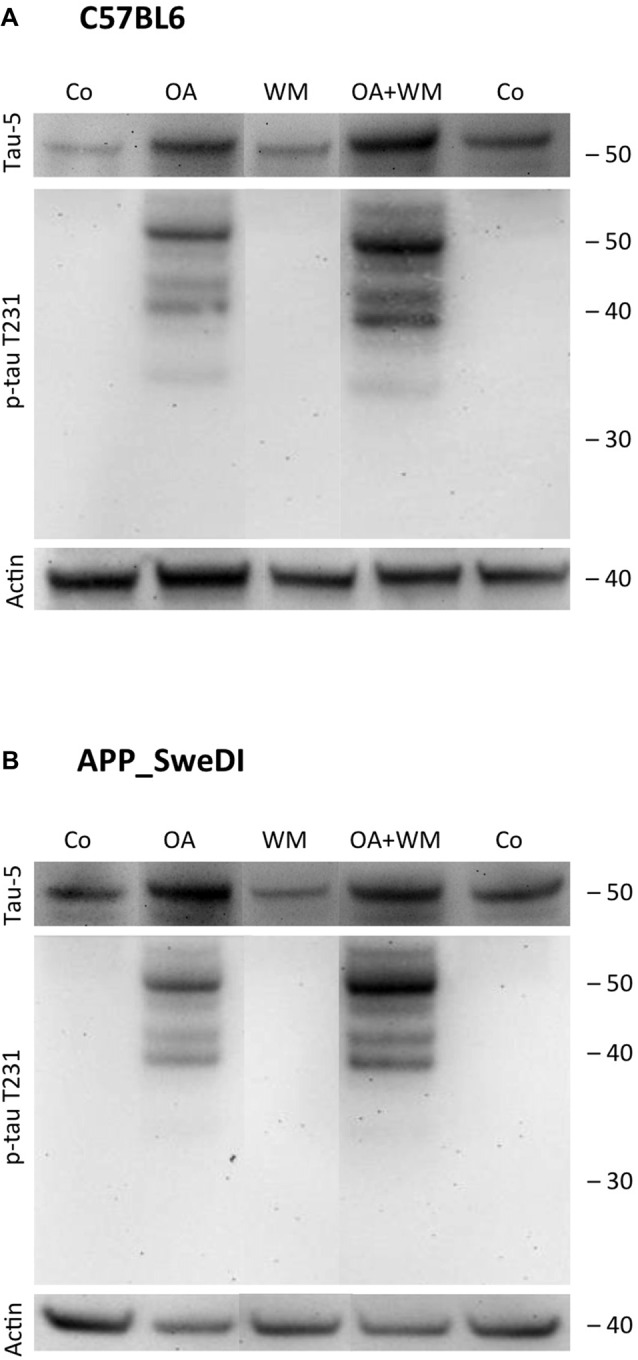
Western blot for p-tau T231. Brain slices of adult WT mice **(A)** or TG mice **(B)** were incubated for 14 days at 37°C without (Co) or with Okadaic acid (OA), wortmannin (WM), or combination OA+WM. Extracted brain slices were subsequently analyzed by Western blot using antibodies against total tau (tau-5) and phospho-tau-T231. Size markers are given as kDa on the right side. Actin served as a control.

**Figure 5:**
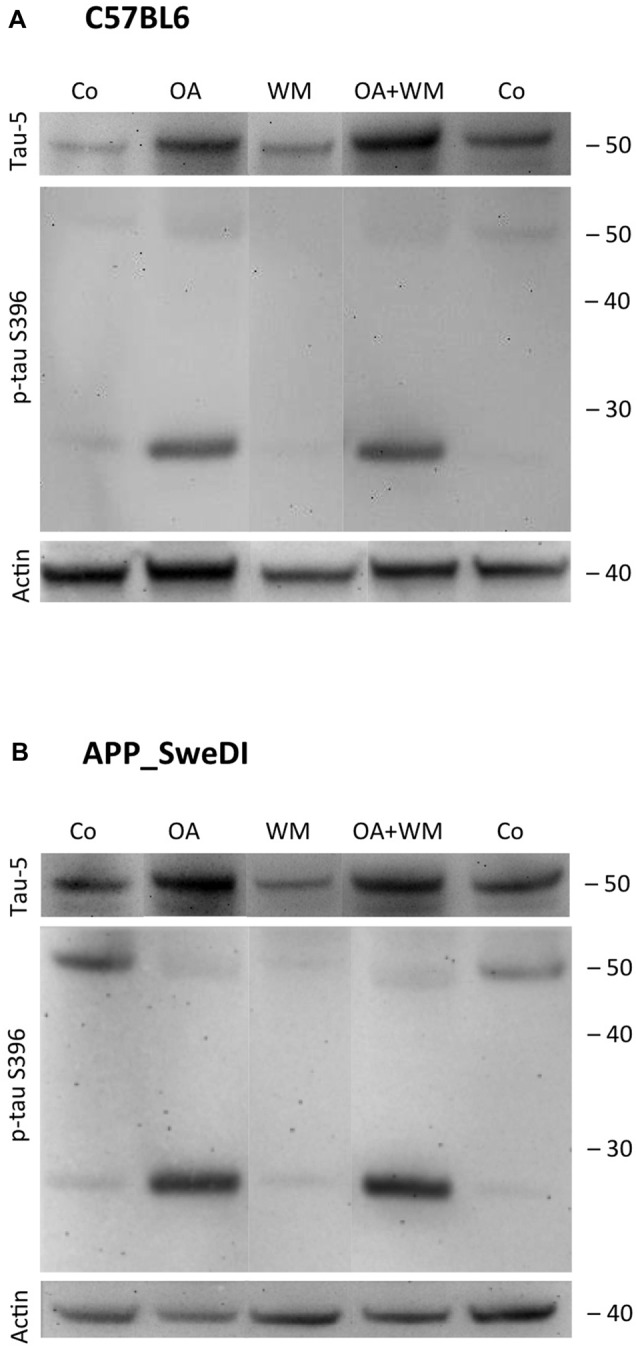
Western blot for p-tau S396. Brain slices of adult WT mice **(A)** or TG mice **(B)** were incubated for 14 days at 37°C without (Co) or with Okadaic acid (OA), wortmannin (WM), or combination of OA+WM. Extracted brain slices were subsequently analyzed by Western blot using antibodies against total tau (tau-5) and phospho-tau-S396. Size markers are given as kDa on the right side. Actin served as a control.

**Figure 6:**
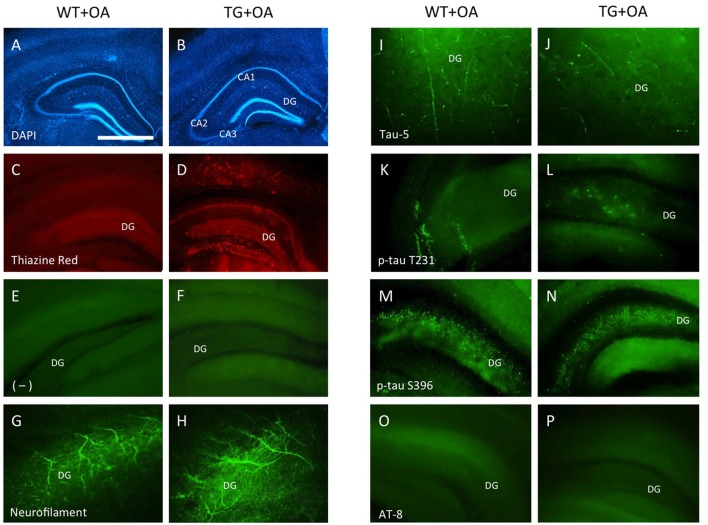
Immunostainings of adult organotypic brain slices at the hippocampal level. Brain slices were prepared from WT or TG adult mice and incubated with okadaic acid (OA). Brain slices were stained for nuclear DAPI (blue, **A,B**) showing intact brain structures. Thiazine Red staining **(C,D)** shows red fluorescent plaques in the hippocampus **(D)** of TG mice, while only background is seen in the hippocampus **(C)** of WT mice. Staining of neurofilament fibers **(G,H)** as well as Tau-5 **(I,J)** shows good neuronal viability both in WT and TG mice. OA induced phospho-tau-T231 in the hippocampus of slices of WT **(K)** and TG mice **(L)** whereas only background was observed in control treated slices **(E,F)**. Phospho-tau-S396 shows a strong immunoreactivity in the hippocampus both in slices of WT and TG mice **(M,N)** after treatment with OA. Neither in WT nor in TG mice the formation of neurofibrillary tangles (NFTs) was seen with AT8 antibody **(O,P)**. Scale bar in **(A)**: 400 μm **(A–D)**, 150 μm **(E,F,M–P)**, 80 μm **(G,H,K,L)**, 40 μm **(I,J)** (DG, dentate gyrus; CA1–3, pyramidal neurons of hippocampus).

### Hyperphosphorylation of Recombinant Tau and Total Tau

As a control recombinant human and mouse tau was hyperphosphorylated with GSK-3β *in vitro*. GSK-3β hyperphosphorylated human recombinant tau at positions tau-S199, tau-T231 and tau-S396 gave a strong band of approximately 60 kDa size (Figure 2). Similarly, also recombinant mouse tau was hyperphosphorylated at all three sites with a size of approx. 50 kDa (Figure 2). Omission of the enzyme or the recombinant proteins gave only background stainings in p-tau T231 and p-tau S396 (Figure 2). Western Blot analysis showed again total tau at the same size (Figure 2).

### Effects of Exogenous Stimuli on WT and TG Mice

Western Blot analysis showed total tau immunoreactivity at a size of 50 kDa both in WT and TG mice. OA and OA+WM caused an upregulation of total tau compared to the control slices in WT mice and in slices treated with OA in TG mice (Figures 3–5; Table 1). Furthermore, there were significant differences for total tau between WT and TG mice in control slices and in slices treated with OA (Table 1). Treatment with OA or OA+WM induced hyperphosphorylation of tau-S199 in the TG mice but not in the WT mice (Figures 3A,B, Table 1). None of the treatments caused hyperphosphorylation of tau-S199 in slices of WT mice (Figure 3A). However, in slices of TG mice, hyperphosphorylated tau protein of 50 kDa size after incubation with OA or OA+WM was seen resulting in significant differences compared to control treatment and to the WT mice (Figures 3A,B, Table 1). Regarding phosphorylation of tau-T231 no effect between TG and WT mice was seen (Figures 4A,B, Table 1). OA and OA+WM significantly hyperphosphorylated tau protein of 50 kDa size and less strong at 38 kDa both in slices of WT and TG mice compared to the controls (Figures 4A,B, Table 1). A strong significant hyperphosphorylation of tau-S396 of 25 kDa size both in WT and TG mice was seen after incubation with OA or OA+WM, however, there was no significant difference between WT and TG (Figures 5A,B, Table 1). Slight bands of 50 kDa size were observed after treatment with OA and OA+WM in WT mice, but in the slices of TG mice there were also proteins of 50 kDa size in the controls visible (Figure 5B). Phosphorylation at tau-S396 did not show differences between the groups at the 25 kDa lane, but differences in control treated slices could be observed between WT and TG mice of a 50 kDa fragment (Figures 5A,B, Table 1).

**Table 1:** Quantitative analysis of total tau and phosphorylated tau fragments in adult organotypic brain slices.

WT	kDa	(-)	OA	WM	OA+WM
Total tau	50	32 ± 4 (4)	86 ± 18 (4)*	34 ± 9 (4) n.s.	91 ± 35 (4)**
p-tau S199	50	3 ± 0 (4)	6 ± 2 (4) n.s.	2 ± 1 (4) n.s.	5 ± 2 (4) n.s.
p-tau T231	50	2 ± 1 (4)	84 ± 14 (4)***	3 ± 2 (4) n.s.	85 ± 25 (4)***
	38	2 ± 1 (4)	46 ± 14(4)**	2 ± 1 (4) n.s.	69 ± 25 (4)***
p-tau S396	50	15 ± 5 (6)	34 ± 19 (6) n.s.	11 ± 3 (6) n.s.	15 ± 3 (6) n.s.
	25	6 ± 1 (6)	106 ± 35 (6)***	10 ± 4 (6) n.s.	84 ± 6 (6)***
**TG**	**kDa**	**(-)**	**OA**	**WM**	**OA+WM**
Total tau	50	68 ± 14 (4)^§^	155 ± 16 (4)**§	30 ± 9 (4) n.s.	93 ± 31 (4) n.s.
p-tau S199	50	1 ± 0 (4)	18 ± 3 (4)**§§	2 ± 1 (4) n.s.	18 ± 2 (4)***§§
p-tau T231	50	1 ± 0 (4)	89 ± 14 (4)***	4 ± 2 (4) n.s.	150 ± 29 (4)***
	38	1 ± 0 (4)	55 ± 14 (4)***	1 ± 0 (4) n.s.	81 ± 20 (4)***
p-tau S396	50	50 ± 17 (6)	27 ± 7 (6) n.s.	19 ± 11 (6) n.s.	24 ± 3 (6) n.s.
	25	10 ± 2 (6)	101 ± 19 (6)***	6 ± 1 (6) n.s.	93 ± 10 (6)***

Organotypic brain slices from adult wildtype mice (WT) and transgenic mice (TG) were incubated without (-), with okadaic acid (OA), wortmannin (WM) or combinations (OA+WM) for 2 weeks and evaluated for tau by Western Blots. Values are given as mean ± SEM optical density (n = number of animals). Background was subtracted and Actin served as a control. Statistical analysis was performed by one-way ANOVA with a subsequent Fischer LSD *post hoc* test. Groups were compared against controls (-): *p < 0.05; **p < 0.01; ***p < 0.001; n.s. not significant. WT and TG mice were also compared: ^§^p < 0.05, ^§§^p < 0.01.

### Immunostainings at the Cellular Level

Strong Thiazine Red plaques (Figure 6D) were found throughout the brains in the TG mice, while no staining was visible in WT mice (Figure 6C). A moderate immunostaining was seen for total tau-5 (Figures 6I,J). Slices treated with OA showed an upregulation of tau-T231 in the hippocampus in WT and TG mice (Figures 6K,L) compared to the controls (Figures 6E,F). OA caused a strong hyperphosphorylation of tau-S396 in slices of WT and TG mice in the hippocampus (Figures 6M,N). The AT8 antibody revealed the absence of NFTs in WT or in TG mice (Figures 6O,P).

### Effects of the NMDA Receptor Antagonist MK801

**Figure 7:**
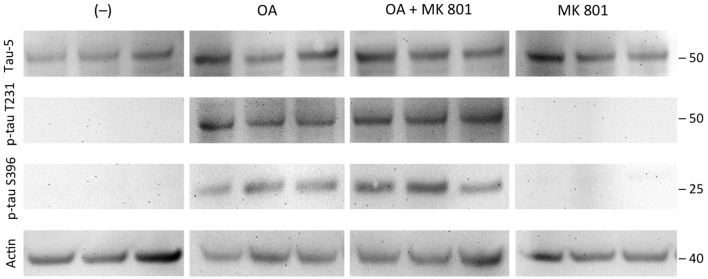
Effects of MK 801. Brain slices of adult TG mice were incubated for 14 days without treatment (–), with okadaic acid (OA), with OA and N-methyl-D-aspartate (NMDA) receptor antagonist MK801 (OA+MK801) or with MK801 alone. Extracted brain slices were subsequently analyzed by Western blot using antibodies against total tau (tau-5), phospho-tau-T231 and phospho-tau-S396. Size markers are given as kDa on the right side. Actin served as a control. Note that the NMDA antagonist MK801 did not counteract OA-induced hyperphosporylation.

Total tau (tau-5) levels were stable independent of the treatment of the slices with OA or OA+MK801 (213 ± 56 OD vs. 156 ± 45 OD, *n* = 3, n.s.; Figure 7). No significant differences were seen in slices treated with OA and OA+MK801 for p-tau T231 (78 ± 28 OD vs. 87 ± 19 OD, *n* = 6, n.s.) or p-tau396 (106 ± 15 OD vs. 136 ± 24 OD, *n* = 6, n.s.). Slices without treatment and with MK801 alone showed no expression of total tau. Actin served as a control (Figure 7).

### Interaction of Plaques and pTau

**Figure 8:**
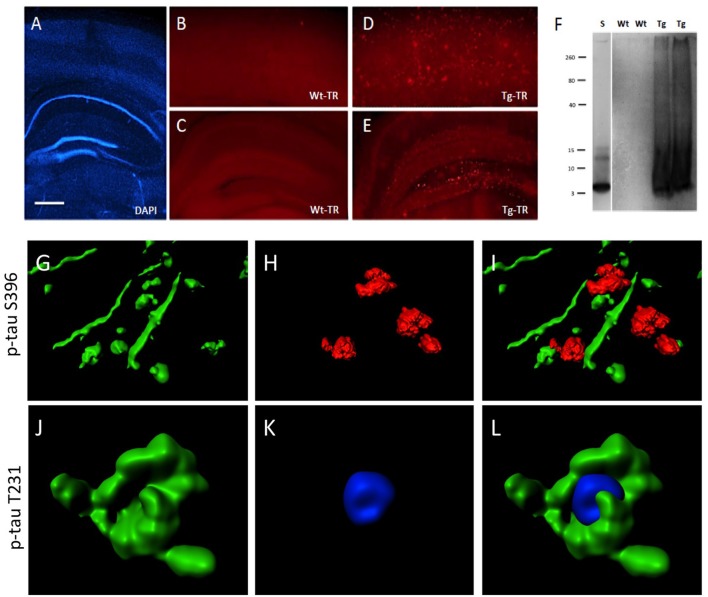
Interaction of beta-amyloid (Aβ) plaques and phospho-tau at the hippocampal level. Brain slices were stained for nuclear DAPI (blue, **A**) showing intact brain structures at the hippocampal level. Thiazine Red staining **(B–E)** shows plaques in cortex **(D)** and hippocampus **(E)** of TG mice, while only background is seen in cortex **(B)** and hippocampus **(C)** of WT mice. A Western Blot shows aggregated Aβ in TG mice but not in WT mice. A standard (S) shows the 4 kDa peptide as a control **(F)**. Confocal microscopy shows p-tau S396 positive staining (Alexa-488, green) in nerve fibers associated with Thiazin red+ Aβ plaques **(G–I)** and p-tau T231 positive staining (Alexa-488, green) in cytoplasm around nuclear blue DAPI **(J–L)**. **(I,L)** show the respective merged pictures. Scale bar in **(A)**: 400 μm **(A,C,E)**, 77 μm **(B,D)**, 25 μm **(G–L)**.

Brain slices cultured from TG mice contained many Aβ plaques in cortex and hippocampus, compared to negative WT mice (Figures 8A–E). A Western Blot confirmed aggregated Aβ in slices taken from TG mice (Figure 8F). In order to quantify the interactions between pTau and plaques, brain slices from TG mice were treated with OA for 2 weeks. Immunoreactivity for p-tau S396 was predominantly found in nerve fibers (6.1 ± 0.4 fibers/field, *n* = 3), was significantly associated with plaques (2.1 ± 0.2 interactions/field, *n* = 3), but was less seen in the cytoplasm (0.3 ± 0.1 cells/field, *n* = 3; Figures 8G–I). In contrast, immunostaining for p-tau T231 was mainly found in the cytoplasm surrounding DAPI+ nuclei (4.5 ± 0.5 cells/field, *n* = 3), but not within nuclei and was also not seen in nerve fibers (0.3 ± 0.1 fibers/field, *n* = 3) and not associated with plaques (0.5 ± 0.2 interactions/field, *n* = 3; Figures 8J–L).

## Discussion

In the present study, we show that OA induces hyperphosphorylation of p-tau S396, tau-T231 and tau-S199 in organotypic brain slices of adult WT and TG Alzheimer mice. We provide a novel model which allows studying Aβ plaques as well as differential tau hyperphosphorylation in organotypic brain slices.

### Phosphorylation of Tau

There is some discrepancy on the role of tau: while it is well known that tau regulates the stability of microtubuli via phosphorylation and subsequent axonal transport (Hanger et al., 2009b; Ward et al., 2012; Luna-Munoz et al., 2013; Wang and Mandelkow, 2016), others suggest that the deletion or overexpression of tau did not affect the axonal transport rates both *in vivo* (Yuan et al., 2008, 2013) or in cultured sympathetic neurons (Tint et al., 1998). However, it is well established that aggregated tau is abnormally hyperphosphorylated in different diseases, including AD, but it is still not clear if phosphorylation is the main activator for aggregation (see also review Goedert et al., 2017). Tau has more than 40 possible phosphorylation sites and the phosphorylation is regulated by several kinases and phosphatases (Miyasaka et al., 2010; Ando et al., 2016). Many kinases have been described to phosphorylate tau, such as e.g., GSK-3 or cyclin-dependent kinase 5 (cdk5) and the most prominent protein phosphatases (PP) are PP2A and PP2B. It is extremely important to identify the phosphorylation sites in the tau protein, as they are either therapeutic or diagnostic targets (Liu and Götz, 2013; Spillantini and Goedert, 2013; Wang and Mandelkow, 2016). Phospho-tau 181 in CSF e.g., is well established as a diagnostic biomarker for diagnosis of AD (Lewczuk et al., 2004; Blasko et al., 2006; Thomann et al., 2009). The phosphorylation at tau-T231 inhibits the ability to bind to microtubuli by approx. 25% (Sengupta et al., 1998). Alonso Adel et al. (2004) showed furthermore that tau-S199 and tau-T231 are one of the most critical phosphorylation sites leading to a transformation from tau into an inhibitory molecule that sequesters normal MAPs. Modification of phosphorylation in tau-T231 or p-tau S396 may play a role in formation of NFT in the AD brain, whereas especially tau-S396-404 seems to be one of the earliest molecular targets in AD (Abraha et al., 2000; Gong and Iqbal, 2008; Hanger et al., 2009a; Mondragon-Rodriguez et al., 2014). In our present study we focused on p-tau S396, tau-T231 and tau-S199, as they are known to play a crucial role in the hyperphosphorylation and eventually in formation of tau tangles.

### Glycogensynthase-Kinase-3β

The kinase GSK-3 is the most important enzyme to induce hyperphosphorylation of tau. GSK-3 is found in two isoforms, GSK-3α and GSK-3β, whereas the latter seems to be the potent kinase that phosphorylates tau *in vitro* and *in vivo* (Sun et al., 2002; Agarwal-Mawal et al., 2003; Liu et al., 2003). GSK-3 is also regulated through the phosphatidylinositol-3-kinase (PI3K) pathway. In order to prove hyperphosphorylation of recombinant tau at the positions tau-S199, tau-T231 and tau-S396 as a positive control, we demonstrate that both human and mouse tau were hyperphosphorylated at all three binding sites by recombinant GSK-3β. In organotypic hippocampal slices Li et al. (2006) showed selective hyperphosphorylation of tau-S396/404 and tau-S199/202 after overactivation of GSK-3β. In our present study (data not shown) we added recombinant GSK-3β to the medium in slices but we could not induce hyperphosphorylation of tau in organotypic brain slices. This could be due to the instability of the enzyme, a rapid deactivation in the medium or a lack of uptake into the cells.

### Wortmannin

WM is an inhibitor of PI3K and can lead to the activation of GSK-3 causing hyperphosphorylation of tau (Li et al., 2006). Indeed Li et al. (2006) succeeded to induce hyperphosphorylation of tau-S396/404 in organotypic brain slices with 10 μM WM, but at the same time WM reduced hyperphosphorylation of tau-S199/202 in the slices. This effect was due to overactivation of GSK-3β. In our experiment we used 10 μM WM as a positive control, but could not induce hyperphosphorylation of tau. These conflicting results may occur due to methodological differences, as we used adult organotypic mouse brain slices and cultured them for 2 weeks with WM, whereas Li et al. (2006) used postnatal rat brain slices and incubated them only for 3 h.

### Okadaic Acid

OA is a cytotoxin and was originally isolated from the black sponge *hallichondria okadaii*. Amongst others, it particularly leads to the activation of GSK-3β, neuroinflammation and oxidative stress (Tachibana et al., 1981; Hanger et al., 2009b; Kamat et al., 2013a; Kamat and Nath, 2015; Baker and Götz, 2016; Zhao et al., 2016). Several *in vivo* and *in vitro* studies showed that OA induces tau phosphorylation and is also able to enhance the neurotoxicity of Aβ (Ahn et al., 2016; Baker and Götz, 2016; Zhao et al., 2016). Kamat et al. (2013b) reported that intracerebroventricular administered OA into rats impaired memory in the morris water maze. In our present study we show for the first time that OA at relatively low concentrations of 100 nM over 2 weeks induced hyperphosphorylation of tau at positions tau-S396 and tau-T231 and only weakly at position tau-S199 in organotypic brain slices.

In this context, Kamat et al. (2013b) examined the role of NMDA after OA induction *in vivo* and found that treatment with the NMDA antagonist MK801 restored PP2A, GSK-3β, tau mRNA and protein expression, Ca^2+^/calmodulin-dependent protein kinase II (CaMKII) and calpain expression. These results indicated that the NMDA receptor may possibly play a crucial role in the OA-induced process of tau hyperphosphorylation *in vivo* (Kamat et al., 2011, 2013b). Thus in the present study we tested if the NMDA receptor antagonist MK801 could block the OA-induced hyperphosphorylation of tau, but our data provide clear evidence that MK801 could not counteract this effect. This might be explained by the assumption that the OA-effect needs an intact glutamate (NMDA) neuronal network or that the NMDA receptors are downregulated in our brain slices.

### Tau Fragments

Interestingly, we observed different fragments of hyperphosphorylated tau for tau-S396 and tau-T231. Three fragments were detectable, a 50 kDa form and a 25 kDa form (phospho-tau-S396) and also a 38 kDa form (phospho-tau-T231). In addition we found larger fragments at a size of 80 kDa and 200 kDa but we could not verify their specificity. While it seems likely that the 50 kDa form reflects the full tau protein (Kim et al., 2016) the smaller 25 and 38 kDa forms could not be identified. In general it is very likely, that tau is posttranslationally modified or that splice variants may create different sized proteins. Indeed, truncation of tau protein has been reported and Zilka et al. (2006) found a 29 kDa size truncated tau protein in the brain and spinal cord of a TG *in vivo* rat model expressing human truncated tau. Further, caspases may play a role in cleavage of tau. In fact a caspase tau-A421 cleaved form (TauC3) has been described, being associated with the formation of NFTs (Rohn et al., 2002; Gamblin et al., 2003; de Calignon et al., 2010; Kim et al., 2016). The expression of this TauC3 in TG mice induced the formation of tau oligomers including memory impairment (Kim et al., 2016). Gamblin et al. (2003) showed furthermore, that caspase induced tau-A421 cleavage leads to a faster formation of NFTs. Indeed in AD, tau is often truncated at its C terminus by the proteolytic cleavage of tau-G391 (Grundke-Iqbal et al., 1986; Novak et al., 1993). In our present study we found for the first time two smaller (25 kDa and 38 kDa) hyperphosphorylated tau-S396 and tau-T231 fragments, but we could not yet identify their function. As we did not see degradation in slices treated without OA or with WM, we conclude that the analyzed tau fragments are highly specific. However it must also be noted that the Western blot did not allow more accurate size measurements.

### The Organotypic AD Slice Model: Aβ and Tau

Organotypic brain slices provide a potent *in vitro* system to study neurodegeneration and also neuroprotection. Typically brain slices are derived from postnatal donors, as they survive also over a longer time period and offer a good morphology (Humpel, 2015a). However, in our study postnatal slices are not useful, because plaques in the TG mice develop not until an age of 5–6 months. We already successfully demonstrated that adult organotypic brain slices provide a powerful tool to study clearance and degradation of Aβ plaques *in vitro*, specifically neprilysin, insulysin and matrix metalloproteinases degraded Aβ plaques in these slices (Humpel, 2015b). In the present study, we provide further proofs that indeed adult brain slices show intact vital brain structures, as seen in a time-dependent expression of neurofilament-200 kDa and total tau, low nuclear PI staining and reduced release of lactate-dehydrogenase. However in Western blot analysis a degradation to a lower 110 kDa size was seen, clearly pointing to some neuronal degradation and metabolic processes in cultured slices.

**Figure 9:**
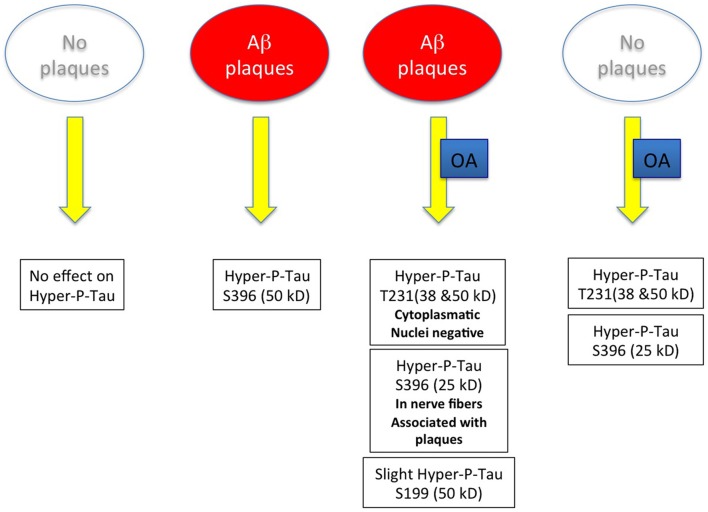
Scheme summarizes the major outcome of this study. Abbreviation: Aβ, beta amyloid; OA, okadaic acid.

As it was our aim in the present study to develop an *in vitro* model for both Aβ and tau pathology, the use of the adult TG mice seemed to be an excellent model to induce tau hyperphosphorylation. In general our data show four major results (see Figure 9): (1) in slices cultured from WT mice without plaques no hyperP-Tau was visible; (2) in slices cultured from TG mice with plaques hyperP-Tau S396 (50 kDa) was induced; (3) OA significantly induced hyper-P-Tau 231 (38–50 kDa) and hyper-P-Tau S396 (25 kDa) independent of the plaques; and (4) a slight activation of hyper-P-Tau S199 (50 kDa) was seen when plaques were present and stimulated with OA. Thus, our data provide more evidence that indeed the hyperphosphorylation of tau of different sites is dependent on divergent exogenous influences. We found that tau-S396 was hyperphosphorylated when plaques were present and independent of exogenous OA. This is an interesting novel finding and in agreement with others, showing that synthetic Aβ_(25–35)_ caused neurodegeneration together with an increased hyperphosphorylation of tau-S396 (Johansson et al., 2006a,b). This clearly confirms the cascade of events that first plaques are developed and then hyperphosphorylation (of tau-S396) can be induced. As a second important finding we found that tau-S199 was only induced when plaques were present and when OA stimulated the slices. This clearly points to co-factors which are necessary to further induce hyperphosphorylation of tau at other sites. As a third novel finding we could show that OA is very potent to induce hyper-P of Tau T231 and S396, independent of the plaques. Further, cleavage of tau-S396 and tau-T231 into smaller 25 kDa and 38 kDa fragments was independent of the presence of plaques but was only induced by OA treatment. In summary, our data provide novel insights on the divergent influence of plaques and exogenous OA treatment on the role of tau.

### Tau Hyperphosphorylation at the Cellular Level and Lack of NFT

In order to proof our findings at the cellular level we performed immunohistochemistry for hyperphosphorylation of tau-S396 and tau-T231. Indeed, we could see strong hyperphosphorylated tau-S396 in the hippocampus both in TG and in WT brain slices. In agreement to our results in the Western blot analysis we observed immunoreactive cells of hyperphosphorylated tau-S396 and tau-T231 in organotypic brain slices. This is partly in agreement with Johansson et al. (2006a), who found strong tau immunoreactivity especially in the dentate gyrus of the hippocampus in rats. Further, we did not see in any cases the formation of NFTs. It is well known that the formation of NFTs is a complex process possibly involving many co-factors such as e.g., inflammation, oxidative stress or ischemia. Also it seems likely that many other phosphorylation sites or processing of tau into high order oligomers must be necessary. However, our study clearly shows that the presence of plaques alone does not induce the formation of NFTs in the presence of hyperphosphorylated tau-S396, tau-T231 or tau-S199.

It was interesting to observe a differential distinct localization of tau-S396 and tau-T231. Immunoreactivity for pTau T231 was mainly found in the cytoplasm surrounding nuclei but almost not in nerve fibers. In contrast, pTau-S396 was located primary in nerve fibers but not in the cytoplasm and was strongly associated with Aβ plaques. Much more work is necessary to confirm if those fibers are of axonal or dendritic origin and how these fibers interact with the plaques. The OA-*ex vivo* model can be a helpful tool to investigate the very early events of tau pathology and the roles of different tau epitopes in various localizations also associated with an existing plaque pathology. Our *ex vivo* data thus support findings that differences in the tau phosphorylation sites are indeed associated with the severity of neuronal cytopathology in AD and it confirms the assumption that an early hyperphosphorylation of tau at specific epitopes may lead to conformational alterations in the pathological cascade (Augustinack et al., 2002).

So far it is not known when and how the pathological cascade is initiated. The “spreading hypothesis” suggests that yet unknown events cause a pathological protein (similar as prions) which start in specific brain areas and spread over the whole brain. This is true for Aβ as well as for tau pathologies. Spreading of aggregated tau along anatomically connected pathways has been reported, but mechanisms are not understood (Liu et al., 2012; Croft et al., 2017). Previous reports indicate that phosphorylation of tau is important in uptake and seeding (Bennett et al., 2017). We show now that OA induced smaller hyperphosphorylated tau fragments (38 and 25 kDa), which is in agreement with Croft et al. (2017). In fact truncated tau or caspase-cleaved tau (tauC3) has been reported to be an important upstream factor in the pathogenesis of NFT in AD (Zilka et al., 2006). Thus more work is needed to evaluate the pathophysiological role of the truncated tau fragments on the tau pathology in AD.

### Limits and Future Strategies

Certainly the study has also some limitations. First of all we examined only three phosphorylation sites whereas more than 40 sites have been identified. Every specific epitope seems to play a different role in the tau cascade and until now the detailed function of every phosphorylation site is still not fully understood. As there are specific antibodies against tau-S396, tau-T231 and tau-S199 and as those sites are reported to play a crucial role in the inhibition of the microtubule binding ability, we decided to investigate those three epitopes. An additional limiting factor is that we could not induce NFTs although we were able to induce hyperphosphorylation.

In conclusion, our model offers a fast and valid way to study both tau and Aβ plaque pathology *in vitro* using our well established AD brain slice model. Plaques and OA differentially induced hyperphosphorylation of tau in these slices. Thus, this novel model provides a strategy for pharmaceutical screening of novel drugs.

## Author Contributions

BMF performed experiments, evaluated data and wrote the manuscript. CH designed the study, planned experiments and wrote the manuscript.

## Conflict of Interest Statement

The authors declare that the research was conducted in the absence of any commercial or financial relationships that could be construed as a potential conflict of interest.
